# Acknowledgement of Reviewers, 2015

**DOI:** 10.5964/ejop.v12i1.1134

**Published:** 2016-02-29

**Authors:** Vlad Glăveanu

**Affiliations:** a EJOP Editor; Aalborg University, Aalborg, Denmark

We are grateful for the reliable and helpful support that we have received from the following colleagues, who provided us with valuable editorial help in the peer review process for *Europe's Journal of Psychology* in 2015:

## Handling Editors

Andrew P. Allen
Ateka Contractor
Constance de Saint-Laurent
Steven Hertler
Maria Kakarika
Maciej Karwowski
Nicholas A. Kuiper
Izabela Lebuda
Natalia Wentink Martin
Rhian Worth

## Reviewers

Andrew P. Allen
Anonymous
Frans Orsted Andersen
Ruggero Andrisano Ruggieri
Gamze Arman Incioglu
Catherine Aubier
Savita Bakhshi Anand
Baptiste Barbot
Claude Belanger
Raoul Bell
Victoria Bellou
Romain Boissonnade
Caterina Bonnin
E. Bora
Belinda Bradley
David Brendel
Stephanie Breux
Damien Brevers
Ronald J. Burke
Carla Canestrari
Flavia Cangia
Miguel Angel Carrasco
Kevin Carriere
Janine Carroll
Pavlina Charalampidou
Ying-he Chen
Russell Clayton
Peter Coleman
Daniel David
Constance de Saint-Laurent
Carolin Demuth
Colin Depp
Nicolas Deuschel
Japinder Dhesi
Jacqueline Dowling
David Dozois

Dorota Dziedziewicz
Ghassan El-Baalbaki
Alexandra Fiocco
Aleksandra Gajda
Panagiotis Gkorezis
Vlad Glăveanu
Susan G. Goldberg
Alessio Gori
Jacek Gralewski
William Peter Hampes
Jennifer Harrison
Steven Hertler
Nicoletta Isar
Dan Ispas
Myrthe Jacobs
Michael Jenkins-Guarnieri
Sian Jones
Maria Kakarika
Pavlo Kanellakis
Maciej Karwowski
Theano Kokkinaki
Zoltan Kovary
Nicholas A. Kuiper
Tiziana Lanciano
András Láng
Izabela Lebuda
Olga Lehmann
Emmanouela Mandalaki
Martínez-Arán
John McCormick
Ricardo Garcia Mira
Melissa Mitchell
Rona Moss-Morris
Alireza Moula
Austin Lee Nichols
Michael P. O'Driscoll
Panayiotis Panayides
Danielle Polage
Beatrice Popescu
Peter Raabe
Joanna Rajchert
Rosana Reis
Giulia Righi
Michelle Roley
Bohdan Roznowski
Stefan Schmertz
Keith Schofield
Jennifer Sheehy-Skeffington
David Silvera
Jonathan N. Stea
Coralia Sulea
Joanna Szen-Ziemianska
Grzegorz Szumski
Ida Toivonen
Taròk Totan
Eirini Tsachouridi
Odin van der Stelt
Martijn van Zomeren
Tony Vernon
Daljinder Ritu Virk
T. Joel Wade
Natalia Wentink Martin
Koa Whittingham
Hedia Zannad
Tomasz Zoltak

